# AQP9 Expression in Glioblastoma Multiforme Tumors Is Limited to a Small Population of Astrocytic Cells and CD15^+^/CalB^+^ Leukocytes

**DOI:** 10.1371/journal.pone.0075764

**Published:** 2013-09-25

**Authors:** Sabina Jelen, Benedicte Parm Ulhøi, Agnete Larsen, Jørgen Frøkiær, Søren Nielsen, Michael Rützler

**Affiliations:** 1 Water and Salt Research Center, Department of Biomedicine, Aarhus University, Aarhus, Denmark; 2 Department of Pathology, Aarhus University Hospital, Aarhus, Denmark; 3 Department of Biomedicine – Pharmacology, Aarhus University, Aarhus, Denmark; 4 The Water and Salt Research Center, Institute of Clinical Medicine, Aarhus University, Aarhus, Denmark; 5 Department of Biochemistry and Structural Biology, Center for Molecular Protein Science, Lund University, Lund, Sweden; University Hospital of Heidelberg, Germany

## Abstract

Aquaporin-9 (AQP9) is a membrane protein channel that is permeable to a range of small solutes, including glycerol, urea and nucleobases. Expression of AQP9 in normal brain is limited, while widespread AQP9 expression has previously been reported in human glioblastoma. However, the specific cellular expression of AQP9 in glioblastoma remains unclear. In this study, we have examined microarrays to corroborate *AQP9* mRNA expression in glioma. These analyses suggested that *AQP9* mRNA expression in glioblastoma is primarily explained by tumor infiltration with AQP9 expressing leukocytes. Immunolabeling confirmed that within tumor regions, AQP9 was expressed in CD15^+^ and Calgranulin B^+^ leukocytes, but also in larger cells that morphologically resembled glioma cells. Specificity of immunoreagents was tested in recombinant cell lines, leukocyte preparations, and sections of normal human brain and liver tissue. Apparent AQP9^+^ glioma cells were frequently observed in proximity to blood vessels, where brain tumor stem cells have been observed previously. A fraction of these larger AQP9 expressing cells co-expressed the differentiated astrocyte marker GFAP. AQP9 expressing glioma cells were negative for the brain tumor stem cell marker CD15, but were observed in proximity to CD15^+^ glioma cells. AQP9 expression may therefore require signals of the perivascular tumor environment or alternatively it may be restricted to a population of glioma stem cell early progenitor cells.

## Introduction

Aquaporin-9 (AQP9) is a member of the major intrinsic protein family. It was originally identified in an expression screen for a putative hepatocyte urea channel [1]. Besides urea, AQP9 was found to be highly permeable to glycerol, adenine and uracil as well as moderately permeable to lactate and β-hydroxybutyrate in the same study [1]. We have recently demonstrated the physiological importance of AQP9 in hepatocyte gluconeogenesis from glycerol [2]. Besides in hepatocytes, AQP9 expression has been described in several tissues, including normal brain. However, the identified locations of AQP9 expression in murine, rat and primate brain were not entirely consistent between studies: AQP9 expression was found in mouse brain in astrocytes, in rat brain tanycytes, ependymal cells, glia limitans and catecholaminergic neurons, as well as in primates in astrocytes and catecholaminergic neurons [3]–[6].

In one study, where knockout mice were used as controls, no specific expression of AQP9 was found in brain, while the protein was readily detectable in liver and epididymis [7]. In a later study, utilizing the same knockout mice, it was concluded that AQP9 is expressed in murine brain, albeit at low levels in a narrow population of neurons [8]. While these discrepancies may in part be explained by species differences, it is not without precedence that immunolocalization studies describe inconsistent observations. Saper [9], [10] has therefore suggested a reasonable set of control experiments that should be conducted in such investigations.

In human glioblastoma, the most common and aggressive type of brain tumor, widespread and enhanced AQP9 expression, compared to normal brain, has been described. These tumors consist of malignant glioma cells, but also of several other cell types including cells of the immune system. Specific cell types that express AQP9 were however not distinguished previously [11]. In support of AQP9 expression in malignant glioma, another group found positive correlation between enhanced AQP9 expression and astrocytoma grade in immunoblots of astrocytoma tissue [12]. The aim in the current study was to verify AQP9 expression in glioblastoma. A rigorous set of control experiments was included. Furthermore, we wished to identify the cellular expression of AQP9 in glioblastoma tissue in co-labeling experiments with antibodies directed to specific cellular markers. We found that AQP9 in glioblastoma tissue biopsies is expressed in a subset of malignant astrocytic cells and in tumor infiltrating CD15^+^ and Calgranulin B^+^ cells, thus identifying these cells as myelomonocytic linage cells, including neutrophils, eosinophils, and some monocytes, but not basophils [13]. We will refer to these cells as myelomonocytic cells throughout the manuscript.

## Results

Previous studies have suggested enhanced AQP9 expression in high-grade glioma [11], [12]. To obtain further evidence for enhanced AQP9 expression in glioblastoma and to understand a possible, underlying mechanism, we analyzed publicly available microarray data sets [14], [15] for correlation between *AQP9* expression and other transcripts. The results are summarized in File S1. We found that *AQP9* mRNA was co-regulated with several transcripts encoding components of the innate immune response, such as complement components and molecules known to mediate responses to bacterial lipopolysaccharide (LPS). Specifically, *AQP9* expression appeared highly correlated with *calgranulin A* and *calgranulin B* expression (other names: *MRP8/S100A8* and *MRP14/S100A9*, respectively). These mRNAs encode proteins forming calgranulin A-calgranulin B dimers, which can act as ligands for the toll-like receptor 4 (TLR4) LPS receptor [16]. Another transcript with strong correlation to *AQP9* expression, *CD14* encodes a LPS receptor [17], [18] and may act as a co-receptor of TLR4 [19]. Calgranulin A and calgranulin B are expressed in monocytes and together account for 40% of the soluble protein in neutrophils [20]. CD14 is highly expressed in monocytes and macrophages and at lower amounts in endothelial and epithelial cells [21]–[23]. No strong correlation between *AQP9* expression and astrocytic markers such as glial fibrillary acidic protein (*GFAP*) and *AQP4* was detected.

**Figure 1:**
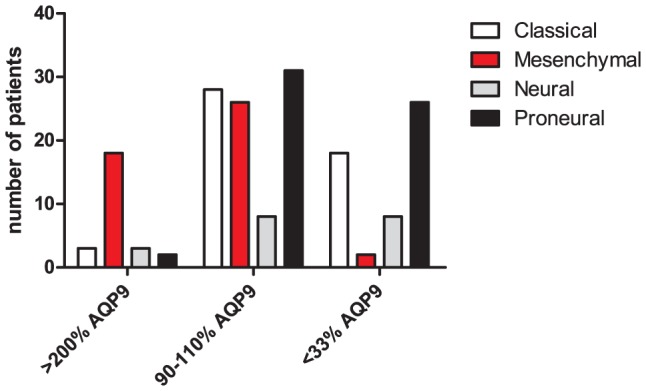
AQP9 mRNA expression levels in four types of glioblastoma. Expression levels of AQP9 in 173 patients were retrieved from the TCGA database and compared to patient descriptors that have previously been associated with four types of glioblastoma: classical, mesenchymal, neural and proneural [25]. Chi-square analysis suggests uneven AQP9 expression levels among these four tumor types (P<0.0001). High AQP9 expression (>200%) appears to be associated primarily with mesenchymal tumors. X-axis labels refer to *AQP9* expression in specific patients compared to average *AQP9* expression in TCGA glioma samples.

In addition, we analyzed *AQP9* mRNA expression in the glioma data set at the “The Cancer Genome Atlas Network” (TCGA, [24]) website. Patient descriptors with low, average and high associated *AQP9* mRNA expression were retrieved. These patient descriptors were compared to a recent report by Verhaak et al. [25], were four types of glioblastoma were distinguished in this data set. We found that *AQP9* expression differed significantly between glioblastoma types (Figure 1). Out of 26 patients with elevated AQP9 expression (more than 2-fold above average), 18 patients were afflicted by mesenchymal glioblastoma. High necrosis and infiltration with inflammatory cells were pointed out as characteristics of mesenchymal tumors by Verhaak et al. (2010) [25]. Furthermore, high expression of *AQP9* mRNA in human leukocytes has been described previously [26]. Therefore, our microarray analyses suggested that previously observed, enhanced *AQP9* mRNA and protein expression in glioblastoma [12] may be explained by tumor infiltration with *AQP9* expressing leukocytes. Alternatively, *AQP9* expression in malignant astrocytic cells might be correlated with leukocyte infiltration.

To distinguish between these hypotheses we investigated the cellular expression of AQP9 in formalin fixed paraffin sections of 10 human tumor biopsies that were characterized as glioblastoma tissue by a pathologist. Initially we sought to establish specificity of the utilized immunoreagents. Among several tested anti AQP9 sera, we found that a serum raised against a carboxy-terminal portion of rat AQP9 (RA2674/685) [6] was the most suitable immunoreagent for our study. Tested antisera and evaluations are listed in Table 1.

**Table 1:** Control experiments performed to assess antigen recognition by antisera and specificity in paraffin embedded tissue sections.

Antibody	Tissue analyzed	Result (labeled structures)
**anti-AQP9** RA2674/685	human liver	hepatocyte basolateral membranes (Figure 2A)
	human leukocytes	PMNs (Figure 2E)
	human brain	negative (Figure S1A)
	U-87 MG-AQP9	a subset of cells (Figure 2C)
	U-87 MG	negative (Figure 2D)
**anti-AQP9** RA2674/685, blocked	human liver	negative (Figure 2B)
	human leukocytes	Negative (Figure 2F)
**anti-AQP9 AD**	U87 MG-AQP9	negative (not shown)
	U-87 MG	negative (not shown)
**anti-AQP9 SC F-17**	U87 MG-AQP9	negative (not shown)
	U-87 MG	negative (not shown)
**anti-AQP9 SC C-18**	U87 MG-AQP9	a subset of cells (not shown)
	U-87 MG	negative (not shown
**anti-GFAP**	human brain	astrocytes (Figure S1B)
**anti-CD133 (3 antibodies)**	human kidney	negative (not shown)
	human placenta	negative (not shown)
	human glioblastoma	negative (not shown)
**anti-nestin**	human glioblastoma	glioma cells (Figure S1C)
**anti-CD31**	human liver	endothelial cells (Figure S1D)
**anti-CD15**	human leukocytes	PMNs (Figure S1 E and H)
**anti-calgranulin A**	human leukocytes	PMNs (Figure S1F)
**anti-calgranulin B**	human leukocytes	PMNs (Figure S1 G and H)

**Figure 2:**
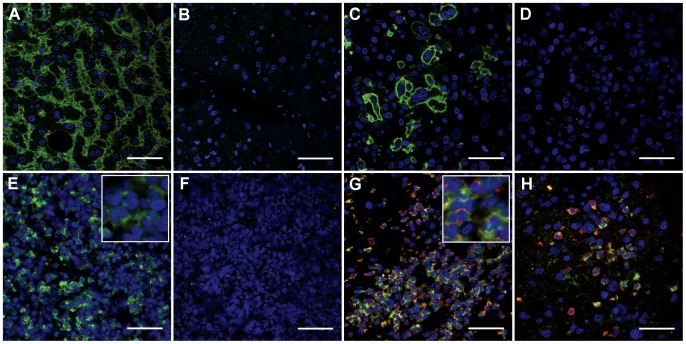
Immunofluorescence labeling of human tissue sections. All sections were labeled with anti-AQP9 RA2674/685 serum (green). A: Liver, B: liver, after AQP9 antibody pre-absorption with immunizing peptide. Labeling of paraffin embedded U-87 MG cells transfected with pIRES-*hAQP9* (C) and pIRES-*hAQP9-inverted* (D) vectors, respectively. (E) Human leukocytes and (F) leukocytes after pre-absorption with immunizing peptide. AQP9 immunoreactivity was observed in a subset of leukocytes that were judged to be primarily granulocytes based on their nuclear shape (inset). (G) Double immunolabeling of AQP9 (green) and CD15 (red) identifying myelomonocytic infiltration in glioblastoma tissue. Cells were labeled by anti-AQP9 and the anti-CD15 myelomonocytic marker (red). (H) Double immunolabeling utilizing anti-AQP9 and anti-calgranulin B (red) identifies myelomonocytic cells in glioblastoma tissue. Scale bars: A–H: 50 µm, inset length E and G: 30 µm.

Control experiments included immunofluorescence labeling of human liver, human leukocytes, normal human brain, and U-87 MG human glioma cells that were transfected to express ectopic *hAQP9*. We note that *hAQP9* transcript was detected in U-87 MG cells by PCR above 30 cycles of amplification. No protein could be detected subsequently by immunofluorescence labeling, as well as in immunoblots (data not shown). We therefore concluded that *AQP9* mRNA and protein expression in native U-87 MG cells is very low. In liver, immunoreactivity of anti AQP9 antibody was detected in hepatocytes (Figure 2A) as previously seen in rodents and humans [6], [7], [27]. Control labeling with pre-absorbed anti AQP9 serum resulted in low background fluorescence (Figure 2B). Similarly, AQP9 immunoreactivity was not observed in normal human brain (Figure S1A). U-87 MG cells transiently transfected with human AQP9-expressing vector showed AQP9 immunoreactivity at the cell periphery (Figure 2C), while there was no immunolabeling of cells that were transfected with a control vector (Figure 2D). In the control vector *hAQP9* cDNA was inserted in 3′ to 5′ orientation, i.e. inverted, downstream of the cytomegalovirus promoter of transcription. Furthermore, we detected AQP9 immunoreactivity in a population of isolated human leukocytes (Figure 2E), which is in agreement with previous findings, where *AQP9* mRNA was detected in human leukocytes [26], [28]. No unspecific labeling was observed in pre-adsorption controls (Figure 2F). Double immunolabeling utilizing anti-AQP9 serum and myelomonocytic markers, anti-CD15, anti-calgranulin A, and anti-calgranulin B, revealed co-localized immunoractivity (Figure S1 E–K), indicating that among circulating leukocytes, AQP9 is expressed in myelomonocytic cells.

In human glioma biopsies we detected a large number of AQP9^+^ cells either scattered throughout tumor parenchyma or in larger clusters that were assessed as immune cells based on cell size. Further double immunolabeling with anti-AQP9 serum along with CD15 (Figure 2G) and calgranulin B (Figure 2H), respectively, confirmed that these cells were myelomonocytic cells. These cells were in part found among erythrocytes, indicating that some of these cells may stem from hemorrhage. We furthermore observed myelomonocytic cells in proximity to and within necrotic tissue areas. Visual impressions of the extent of AQP9 and CD15 immunolabeling are summarized in table 2.

**Table 2:** Semiquantification of AQP9, CD15, GFAP and AQP4 expression and co-expression, respectively.

Patient #	AQP9+ glioma cells	CD15+ glioma cells	AQP9+ CD15+ glioma cells	GFAP+ tumor cells	AQP9+ of GFAP+ cells	CD15+ cells area (outside blood vessels)	AQP4
1	≤5%	≤0.1%	0%	≤80%	≤50%	25% area, high number	50%
2	≤5%	0%	0%	≤80%	≤50%	20–40% area in necrotic area	≥95%
3	0%	0%	0%	≤80%	0%	few cells	N.Q.
4	≤0.1%	0% (*)	0%	≤90%	too few to quantify	few cells, near vessels	≥70%
5	≤5%	≤1%	0%	≤90%	>50% (but weak GFAP in AQP9+)	scattered single cells	N.Q.
6	≤1%	≤1%, very faint labeling	0%	≤40%	0%	scattered clusters of 5–10 cells, some bigger clusters	100%
7	0%	≤1%, very faint labeling	0%	100%	0%	≤1% area, very few cells	100%
8	≤5%	0%	0%	≤50%	≤20%	80% area in big necrotic chunk	≥95%
9	≤5%	0%	0%	≤30%	≤5%	vast in necrotic area, few outside	100%
10	0%	0% (*)	0%	N.Q.	N.Q.	0%	≥95%
11	N.Q.	N.Q.	N.Q.	N.Q.	N.Q.	N.Q.	100%
12	N.Q.	N.Q.	N.Q.	N.Q.	N.Q.	N.Q.	100%
13	N.Q.	N.Q.	N.Q.	N.Q.	N.Q.	N.Q.	100%
14	N.Q.	N.Q.	N.Q.	N.Q.	N.Q.	N.Q.	≥95%
15	N.Q.	N.Q.	N.Q.	N.Q.	N.Q.	N.Q.	100%

**N.Q., not quantified due to poor tissue quality.**

(*) A population of weakly CD15^+^ astrocyte-like shaped cells (∼10% of cells) was observed.

**Figure 3:**
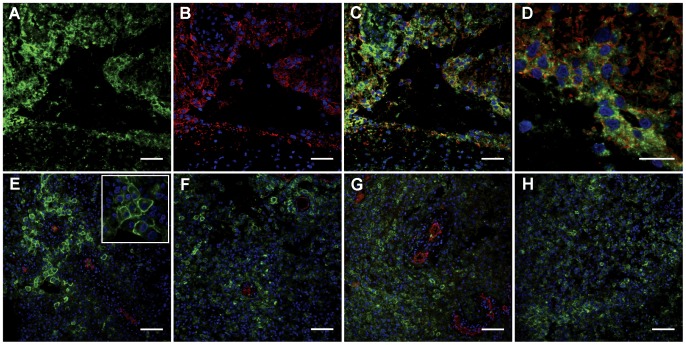
Immunofluorescence labeling of human glioblastoma tissue sections. A–D: Double immunofluorescence labeling utilizing anti-AQP9 RA2674/685 (green) and anti-GFAP sera (red). Partial overlap between AQP9 and GFAP immunolabeling was observed in apparent astrocytic cells. E–H: Double immunofluorescence labeling utilizing anti-AQP9 and anti-CD31 (red) sera. AQP9 immunoreactivity is localized at the periphery of malignant cells, which is consistent with expected plasma membrane labeling of this channel protein (E inset). In many places AQP9-postitive tumor cells are localized near blood vessels (E–G). However, some clusters of AQP9-positivie cells were found without obvious proximity to blood vessel (H). Scale bars: A–C: 50 µm, D: 25 µm, E–H: 100 µm, inset width E: 120 µm.

In addition to anti-AQP9 labeling of myelomonocytic cells we found AQP9 immunoreactivity in cells that morphologically resembled astrocytic tumor cells. These cells were typically larger (16.6±3.1 SD µm apparent diameter on sections) than AQP9-positive myelomonocytic cells (10.9±3.0 SD µm apparent diameter on sections). Such cells were observed in 7 out of 10 patient biopsies analyzed. Antibodies directed to GFAP are frequently used in the diagnosis of astrocytic tumors. Consistent with expectations, GFAP was widely expressed in the studied tissue sections, while it was lost in some areas [29], [30]. Double immunolabeling, utilizing anti-GFAP and anti-AQP9 sera, showed that some AQP9-positive cells co-expressed GFAP (Figure 3A–D) thus identifying these cells as astrocytic cells. The fraction of AQP9 and GFAP double-labeled cells varied between patients. A semi-quantification of apparent co-localization is given in Table 2. We note that GFAP expression frequently appears weak in AQP9 expressing cells. Furthermore, we semi-quantified the level of another related water channel, AQP4 in the investigated biopsies and found widespread expression in all patient biopsies analyzed for this marker (Figure S1L). AQP4 expression was observed in 50–100% of glioma cells (Table 2).

We found that larger AQP9 expressing cells were not uniformly present throughout tumor biopsies. In several places, these AQP9^+^ cells were apparently localized close to blood vessels. AQP9 immunoreactivity around blood vessels was confirmed in co-labeling experiments with anti-AQP9 and anti-CD31 sera (Figure 3E–G). CD31 is specifically expressed in endothelial cells [31]. No overlap between AQP9 and CD31 immunoreactivity was observed, indicating absence of AQP9 from endothelial cells. In addition, we found aggregates of larger AQP9 positive cells in regions without visible blood vessels (Figure 3H). Due to the sometimes moderate quality and limited amounts of available tissue, we cannot exclude that blood supply was present in the vicinity of these clusters or in nearby sections.

**Figure 4:**
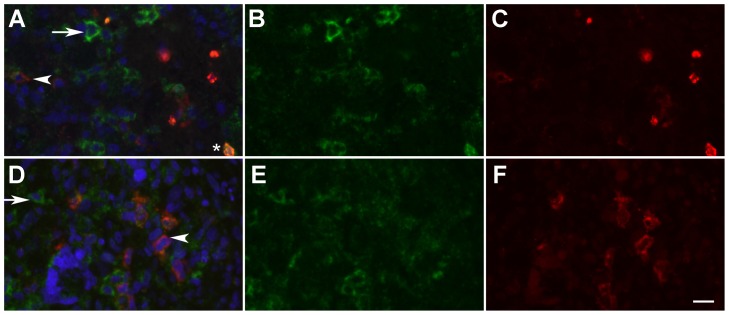
Double-immunofluorescence labeling of human glioma tissue. Robust anti-CD15 (red) and anti-AQP9 co-labeling was observed in myelomonocytic cells (asterisk). In 4 patient-biopsies weaker CD15 immunolabeling was also observed in a number of larger cells, likely identifying these cells as BTSCs (examples marked by arrowheads). Non-myelomonocytic AQP9 positive cells (examples marked by arrows) were located near putative BTSCs, but no clear examples of AQP9 and CD15 co-expression were observed in these cells. Two areas are shown as overlay images (left), green channel (center) and red channel (right). Nuclei (blue). Scale bar  = 20 µm.

It has been demonstrated that the perivascular environment harbors and maintains a pool of brain tumor stem cells (BTSC) [32]. We therefore reasoned that perivascular AQP9 expressing cells may include BTSCs. In order to test this hypothesis we performed immunolabeling with previously used BTSC markers [33], [34]. Unfortunately, we were not able to obtain satisfactory positive control immunolabeling with available antibodies against CD133 on paraffin embedded human kidney tissue, human placenta, and human glioblastoma tissue (data not shown). In contrast, anti-nestin labeling resulted in widespread immunoreactivity in most tumor areas (Figure S1C). Therefore, in agreement with several previous studies [35]–[37], we concluded that nestin is not a selective marker of BTSCs but is also expressed in a large number of progenitor cells. Furthermore CD15 has been identified as a marker of BTSCs [38], [39]. In addition to robust CD15 immunolabeling of myelomonocytic cells, we observed clear but weaker immunolabeling of larger cells in sections obtained from one patient biopsy. Very faint, CD15 immunolabeling of similar cells was also present in sections obtained from three other biopsies. No clear overlap between CD15 and AQP9 immunolabeling was observed in these cells. However, AQP9^+^ cells were located in proximity to CD15^+^ cells (Figure 4).

## Discussion

Here we describe a detailed immunolocalization of AQP9 in human glioblastoma, along with specific cellular markers. In contrast to a previous study [11], we did not observe ubiquitous expression of AQP9 in glioblastoma biopsies. We found however that in some patient-biopsies anti-AQP9 immunolabling was associated with a large number of myelomonocytic cells. AQP9 immunolabeling was also detected in myelomonocytic cells isolated from a healthy human donor. This finding is in line with previous studies where AQP9 mRNA was identified in human leukocytes [26], [28] and AQP9 protein in rat leukocytes [6]. Furthermore, expression of AQP9 in myelomonocytic cells is consistent with our microarray data analysis where we observed a significant correlation between expression of *AQP9* and transcripts that are typically expressed in leukocytes. Particularly, we detected high correlation between *AQP9*, *calgranulin A* and *calgranulin B* expression. *Calgranulins A* and *B* encode for proteins that account for 40% of the soluble proteins in neutrophils. This correlation at the transcript level was confirmed by co-immunolocalization of calgranulin B and AQP9 in tumor-associated myelomonocytic cells, which include neutrophils. We also found that high levels of *AQP9* mRNA expression were correlated with the mesenchymal type of glioblastoma [25]. This type of glioblastoma is associated with poor prognosis and high levels of inflammatory infiltrates [40]. Correlation of *AQP9* mRNA expression with the mesenchymal type of glioblastoma therefore appears to result at least in part from the correlation of mesenchymal glioblastoma with inflammatory infiltrates. Furthermore, we found that AQP9 is expressed in a subset of larger cells that in part co-express GFAP. We interpret these cells as malignant astrocytic cells. Compared to the number of myelomonocytic cells in biopsy tissue, the number of these apparent astrocytic AQP9 expressing cells was limited. We therefore expect that the influence of these cells on overall transcript levels in microarray studies is equally small. Furthermore this means that AQP9 expression in astrocytic glioma cells, as observed in 7 out of 10 evaluated patient biopsies, may not be correlated with the mesenchymal type of glioblastoma.

Interestingly, in several places AQP9 expressing glioma cells were localized in close proximity to blood vessels. A perivascular niche that maintains brain tumor cells in a self-renewing state, so called brain tumor stem cells (BTSC) has been described previously [32], [41]. In a recent study AQP9 expression has been described in human glioma derived neurospheres [42], which suggested AQP9 expression in BTSCs. Widely used BTSC markers were in our hands not applicable: While we were unable to detect the CD133 antigen in paraffin embedded tissue sections, immunolabeling using an antibody directed to nestin resulted in widespread tumor cell immunoreactivity. The latter was not in agreement with the idea that nestin could be used as a marker for BTSCs. It has been reported recently, that CD133 is only expressed in a subset of glioblastomas [43], while observation of abundant nestin expression is in complete agreement with several previous reports that came to the same conclusion [35]–[37]. Furthermore, specific CD15 immunoreactivity on BTSCs has been described by several groups [38], [39], [44], [45]. We did observe a small number of apparent CD15^+^ cells that were judged to be astrocytic glioma cells, based on size. We did not observe co-expression of AQP9 and CD15 in these cells. However, CD15^+^ cells were observed next to AQP9^+^ cells. The number of patients and observed CD15^+^ cells analyzed in this study was however too small to draw general conclusions based on these observations. Furthermore CD15^+^ BTSCs are not present in every glioblastoma brain tumor and no single marker protein is currently known that can conclusively be linked to a glioblastoma stem cell phenotype [46].

## Conclusions

Our studies identify AQP9 expression in a limited population of glioma cells as well as in tumor infiltrating myelomonocytic cells. AQP9 expression within tumor parenchyma is not widespread, compared to abundant expression of the related water channel AQP4. Consequently, we reason that a major effect of AQP9 expression on tumor associated edema is unlikely. AQP9 expression in astrocytic cells near blood vessels suggests a connection to signals of the perivascular environment. Alternatively, these cells may represent a population of early glioma stem cell progenitor cells, based on observed proximity to CD15^+^ cells. A putative role of AQP9 in myelomonocytic cells in general as well as with regard to glioblastoma remains to be determined.

## Materials and Methods

### Analysis of microarray data

Microarray data sets E-MEXP-567 (16 patients) and E-GEOD-4290 (178 patients) were downloaded at the EBI website (http://www.ebi.ac.uk/), imported into Microsoft Excel and the data headers were edited to fit the Clustal 3.0 input format. Clustal [47] version 3.0 was downloaded at http://bonsai.hgc.jp/~mdehoon/software/cluster/. Treeview was downloaded at http://jtreeview.sourceforge.net. E-MEXP-567 data were filtered in the software so that only genes with an expression value of 10 in at least 10 samples were considered (15677 probe sets out of 21587 fit the criteria). The same list of 15677 probes was selected with the help of a macro in Microsoft Excel before a separate analysis of the E-GEOD-4290 data set. Genes were normalized and centered to the median, before performing complete linkage hierarchical clustering. The resulting files were imported into Java TreeView and clades containing *AQP9* and related probe expression values were exported as a text files. After calculation of Pearson correlation coefficients of expression similarity between *AQP9* and related probe sets, utilizing Graphpad Prism 5 software, genes corresponding to these probe lists were retrieved and analyzed utilizing DAVID [48], [49]. The top ranked genes in both lists appeared similar. A more detailed analysis was conducted for the E-GEOD-4290 data set, which is summarized in Spreadsheet S1. Probe to transcript specificity was analyzed with the help of the AffyProbeMiner software [50].

### Tissue preparations

Approval to utilize previously established brain tumor biopsies in this study was obtained from The Central Denmark Region Committee on Health Research Ethics. Tissue was obtained during autopsy. Informed consent was therefore not required under Danish legislation. Data were analyzed anonymously. Human leukocytes were isolated from a blood sample of the senior author (MR), with written informed consent. All tissues were fixed in 10% neutral buffered formalin and sectioned subsequent to paraffin embedding. Some sections were stained with hematoxylin and eosin for tumor grading by a pathologist. Human leukocytes were isolated by a dextran sedimentation method [51]. A partially purified leukocyte pellet was fixed with 10% neutral buffered formalin for 16 hours, dehydrated and processed for paraffin embedding.

### U-87 MG human glioblastoma cells

Human *AQP9* was amplified from cDNA derived from U-87 MG cells utilizing the following oligonucleotides: KpnhAQP9F 5′-AAAGGTACCGTCCTCAGAGAAGCCCCAAG-3′ and NothAQP9R 5′- AAAGCGGCCGCTGACTGCAAATCCAGAGCTGA-3′. The reaction product was inserted into the PCRII-TOPO vector (Invitrogen), sequenced, liberated with *EcoRI* and re-ligated with *EcoRI* digested pIres2-DsRed2 vector (Clontech). Plasmid Vectors containing *AQP9* in 5′-3′ (pIRES-*hAQP9*) as well as 3′-5′ (pIRES-*hAQP9-inverted*) orientation, respectively, were isolated. U-87 MG cells (LGC standards) were cultured under standard conditions and transfected with *hAQP9* vectors, utilizing Lipofectamin 2000 (Invitrogen) according to the manufacturer's specifications. Cells were harvested after they reached confluency, washed in phosphate buffered saline (PBS), centrifuged and fixed in 10% buffered formalin for 16 hours, before paraffin embedding.

### Antibodies

Rabbit anti-rat AQP9 serum (RA2674/685) was raised against a peptide corresponding to amino acids 274–295 and has been described previously [6]. The following commercially available antibodies were used in this study: rabbit polyclonal anti-rat AQP9 (Alpha Diagnostic #AQP91-A); goat polyclonal anti-human AQP9 (F-17, Santa Cruz #sc-14988); goat polyclonal anti-human AQP9 (C-18, Santa Cruz #sc-14989); monoclonal mouse anti-CD31 (clone: JC70A; Dako #M0823; 1∶25); mouse monoclonal anti-CD15 raised against purified neutrophils of human origin (clone: C3D-1; Santa Cruz #sc-19648; 1∶25); goat polyclonal anti-Calgranulin A raised against a peptide mapping at the C-terminus of human origin (C-19; Santa Cruz #sc-8112; 1∶300); goat polyclonal anti-Calgranulin B raised against a peptide mapping near the C-terminus of Calgranulin B of human origin (C-19; Santa Cruz #sc-8114; 1∶600); goat polyclonal anti-GFAP raised against a peptide mapping at the C-terminus of GFAP of human origin (C-19; Santa Cruz #sc-6170; 1∶50); goat polyclonal anti-CD133 raised against a peptide mapping at the C-terminus of CD133 of human origin (C-19; Santa Cruz #sc-19365); mouse monoclonal anti-CD133/1 (clone AC133; Miltenyi Biotec #130-090-422); mouse monoclonal anti-CD133/2 (clone 293C3; Miltenyi Biotec #130-090-851); mouse monoclonal anti-Nestin (clone 10C2; Millipore #MAB5326; 1∶1600). Secondary antibodies: donkey anti-mouse Alexa 555 conjugated; donkey anti-goat Alexa 546 conjugated (Invitrogen) and Donkey anti-rabbit HRP conjugated (Pierce).

### Immunofluorescence

Sections were rehydrated in a series of alcohols (99%, 96%, 70%) for 1 hour, blocked for endogenous peroxides with 3.5% H_2_O_2_ in methanol. Microwave target retrieval treatment was performed with Retrievit TM Target Retrieval Solution pH10 (BioGenex) according to the manufacturer's protocol. Human and mouse tissue sections were blocked with 5% normal human serum and 5% mouse normal serum, respectively. Primary antibodies were diluted in PBS containing 0.3% Triton X 100 (PBST) and 1% serum and incubated with sections for 1 hour at RT, followed by incubation at 4°C overnight. Before incubation with secondary antibodies, sections were washed in 0.1% PBST and blocked with 5% Normal Donkey Serum (NDS). Secondary antibodies and the nuclear counter stain (ToPro, Molecular Probes) were diluted 1∶1000 in 0.3% PBST, 1% NDS and incubated for 1 hour at room temperature with the sections. Tyramide Signal Amplification (TSA) Plus biotin kit (PerkinElmer) was used along with anti-AQP9 (RA2674/685) antibody. Sections were mounted in glycerol mounting medium (Dako) including DABCO (1, 4-Diazabicyclo[2.2.2]octane; Merck Eurolab A/S). Images were acquired on a Leica TCS-SP2 confocal microscope (Heidelberg, Germany). Fluorescence signals were captured within the dynamic range of signal intensity. Images presented in Figure 4 were captured on a Zeiss Axioplan 2 microscope equipped for epifluorescence imaging (Zeiss, Germany) and processed with AxioVision 4.8 software. Images were assembled in Adobe Photoshop 12.0, without adjustments in contrast.

### Statistical analyses

Chi-square analysis was conducted in Graphpad Prism 5.

## Supporting Information

Figure S1 Immunofluorescence labeling of paraffin embedded tissues. In normal human brain, no immunoreactivity of anti-AQP9 (green) was detected (A) and anti-GFAP (red) antibody labeled cells with astrocytic morphology (B). In human glioblastoma tissue, anti-nestin (red) labeling was detected in a large number of glioma cells (C). In human liver, anti-CD31 labeling (red) was restricted to endothelial cells, as judged by morphological appearance (D). E–K: immunofluorescence labeling of normal human leukocytes. E–G and I–K as corresponding magnifications of above panels: Immunoreactivity of anti-AQP9 (green) co-localized with anti-CD15 (E and I), calgranulin A (F and J), calgranulin B (G and K) (all in red) in myelomonocytic cells. H: immunoreactivity of Calgranulin B (green) co-localized with anti CD15 (red). L: AQP4 immunoreactivity (red). Scale bar: A–H, L 50 µm, I–K 10 µm, inset width D 30 µm.
(TIF)

File S1 Analysis of publicly available microarray data sets for correlation between *AQP9* expression and other transcripts. *AQP9* mRNA was co-regulated with several transcripts (ranked by Pearson r) encoding components of the innate immune response, such as complement components and molecules known to mediate responses to bacterial lipopolysaccharide (LPS). Specifically, *AQP9* expression appeared highly correlated with *calgranulin A* (S100A8) and *calgranulin B* (S100A9).
(XLSX)
