# Supplementary figures and images for: AQP9 Expression in Glioblastoma Multiforme Tumors Is Limited to a Small Population of Astrocytic Cells and CD15+/CalB+ Leukocytes
Source: PLoS One. 2013 Sep 25;8(9):e75764. doi: 10.1371/journal.pone.0075764 (PMC3783410; doi:10.1371/journal.pone.0075764)

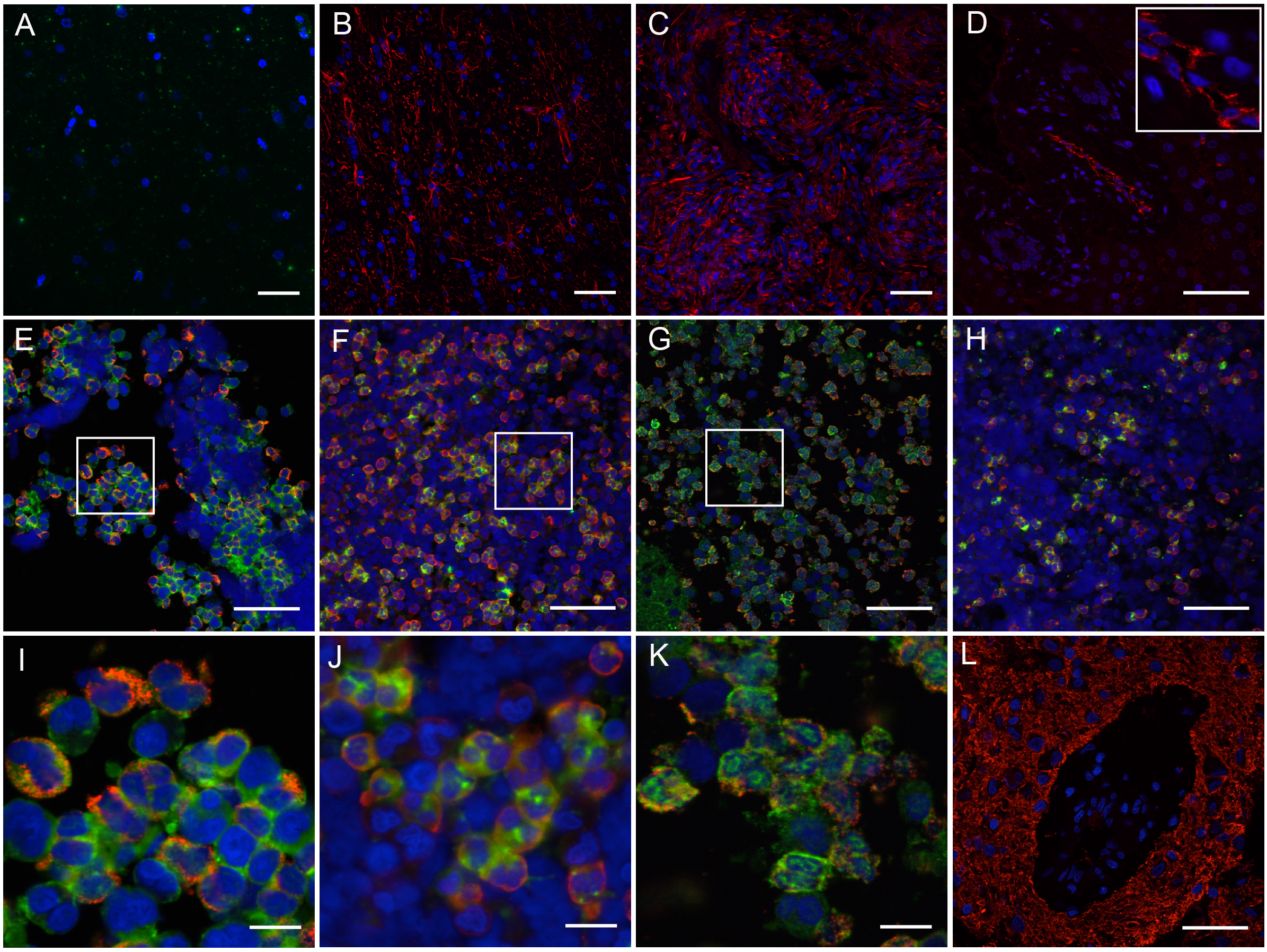

Supplement: Figure S1 — Immunofluorescence labeling of paraffin embedded tissues. In normal human brain, no immunoreactivity of anti-AQP9 (green) was detected (A) and anti-GFAP (red) antibody labeled cells with astrocytic morphology (B). In human glioblastoma tissue, anti-nestin (red) labeling was detected in a large number of glioma cells (C). In human liver, anti-CD31 labeling (red) was restricted to endothelial cells, as judged by morphological appearance (D). E–K: immunofluorescence labeling of normal human leukocytes. E–G and I–K as corresponding magnifications of above panels: Immunoreactivity of anti-AQP9 (green) co-localized with anti-CD15 (E and I), calgranulin A (F and J), calgranulin B (G and K) (all in red) in myelomonocytic cells. H: immunoreactivity of Calgranulin B (green) co-localized with anti CD15 (red). L: AQP4 immunoreactivity (red). Scale bar: A–H, L 50 µm, I–K 10 µm, inset width D 30 µm. (TIF) [file pone.0075764.s001.tif]
